# The Panaritium (Felon)—Consequences and Treatment

**Published:** 1872-11

**Authors:** Carl Proegler


ARTICLE II. *The Panaritium (Felon)—Consequences and Treatment.* By CARL PROEGLER, M.D.

(Continued from page 530.)

It is well known that the felons of the thumb and little finger are the most dangerous, because the flexor pollicis longus has a long sheath which branches out to the root of the hand under the ligamentum carpi volare transversum, and the flexor digiti minimi shows, not always but generally, a communication with the great sheath of tendons of the root of the hand. But even here suppuration does not always stop, reaching often the fore-arm, and in such cases even life is endangered. Unhealthy granulations, thrombosis of veins, septicæmia and pyæmia cause death.

This picture is not merely a phantasia, but exists in reality, and I myself had occasion to witness two cases of this kind in the surgical wards of Berlin; and Huetter very truly says, in a lecture: "I do not wish that in your later praxis you will get acquainted with the panaritium as a disease leading to death, but I am bound to show you also this characteristic phenomenon of the panaritium, and you must never forget that a felon in its consequences may cause death and really has caused it." Therefore the young and inexperienced reader ought to be on his guard, and make himself thoroughly acquainted with the disease under discussion.

Periostitis is very often caused by the panaritium, and taking into consideration the narrowness of the parts, (the flexor tendon and the periosteum of the phalanges are near together) the posterior wall of the sheath of the tendon forms with the periosteum an almost united tissue, and it is therefore self-evident that every suppurative periostitis of the sheath of the tendon and every suppurative synovitis of the latter endanger the periosteum. Clinical experience shows this abundantly.

We very seldom see suppurative periostitis or suppurative synovitis of the sheath of the flexor tendons running alone; they are generally combined, as already explained. Suppurative periostitis is always a disagreeable complication of the panaritium on account of the neighborhood of the sheath of the flexor tendon, or by following necrosis, but here another danger lurks behind—the danger to the articulations which are lying nearest to the diseased phalanges. Pus finds its way into the small synovial cavity of the phalangeal articulation, and besides necrosis of bone, we have the consecutive state of a suppurative articular inflammation, articular fistulæ, necrosis of cartilage, resorption of cartilage, contraction, and, lastly, anchylosis of the limb.

The reader may ask, What is the bearing of all these specified phlegmonous diseases, arising from the panaritium, upon the function of the fingers? What prognosis can we give?

Prognosis is very bad for the function, but it is rather difficult to picture the manifold and grave functional disturbances which remain after these processes.

Paratendinous phlegmonous inflammation leads either to necrosis of the flexors, or to a gluing and growing together of the flexors with the sheaths and the paratendinous connective tissue. This process saves the patient the exfoliation of the necrotic sheath, and the public at large looks upon this as a normal process and gives it the name of "worm." It does not make any difference whether the tendon is forced out, or whether it grows together with the surrounding tissue. In the first case, the contractile substance of the muscles is wanting, and consequently cannot move the bone; in the latter case, the phalanges, which ought to be moved, remain stationary. It tears only on the skin and connective tissue. Movement of the anterior phalanges is entirely destroyed.

Stiffness of a finger or articulation amounts to a good deal. The hand is the first moving apparatus of the whole body; one sheath, one articulation less, causes a deficiency in its working. Take for instance the case of the middle finger glued together with the tensors by suppurative inflammation of the flexor tendons. Not only active movement, but even flexure of the finger is destroyed. The shortened tissue on the volar surface cannot follow the stretch of the flexing muscle, and so is flexure of the root of the hand also impaired.

Flexing and stretching of the hand in the metacarpus induces a stiffness of the finger tendons surrounding this articulation. If the movability of one is impaired, movement of the root of the hand is also, and therefore flexing and stretching of the metacarpal articulation cannot properly be done.

In these articulations lies the whole secret of finger movement. It is a unit; if one suffers, all suffer.

The treatment of the panaritium is very simple, if properly and early done. The sovereign remedy for the *initial state and florescence* is *incision*. It ought to be done early, and in such a manner that the whole panaritial growth will be entirely destroyed. It ought at the beginning to be not larger than a few lines, because primary inflammation is not so extensive. Injuries of other organs are not possible because the greater nerves and arteries on the volar surface are very seldom touched on account of the more central position of the panaritium, and even if cut, it is of no importance. The incision ought to be made lengthwise, so as to avoid possible injuries to vessels, etc.

Once in a while it may occur that the *arcas volaris sublimis* is cut, but then the cut ought to be deep, so as to ligate the two vessels.

It is of importance in a rational treatment of the panaritium that the incision be made early. Surgeons ought to emancipate themselves and the laity from the absurd idea that on the fingers or other places, suppuration ought *to get ripe first*. Poultices, salves and other humbug remedies will give rise to necrosis of tendons, a gluing together of tendons, and even contractions of articulations,—in short, useless hands and fingers.

At the beginning of a panaritium there is but little to be detected by fluctuation or other examinations. Huetter, whom I follow, takes a fine probe and touches the central part of the panaritial swelling, asks the patient where he feels by this first palpation the *most intense pain*, and makes there the incision. This point covers in the beginning of the panaritium not more than a quarter of a line, but if taken for the incision no one will be misled.

Huetter says, very correctly, that it may seem insignificant, but he asserts that he never made a useless incision, and every surgeon of experience will agree with him. By early incision, lymphangitis, lymphadenitis, fever, etc., are avoided. Tendons, periosteum and joints are saved from suppuration, and therefore early incision is a real blessing.

After treatment is very simple. A little carbolic acid, warm baths morning and night for the hand, etc., are all that is necessary.

Suppurative inflammation of the tendons, periostitis, and inflammation of joints may be yet properly treated, but the results are sometimes not very brilliant.

The sheath of the tendon ought to be cut lengthwise; suppuration will come to a standstill, and nourishment of the tendons be saved. After sequestra have been removed, passive motion should be instituted, but I warn practitioners *against the use of straight splints* for the finger; stiffness of the finger is always the result. If splints are used, they ought to be curved, and sometimes by this course we may succeed in getting a tolerably good finger for the patient.

Contractions of joints and anchyloses, which follow perforation of pus into the capsular ligaments, may be corrected on general surgical principles; resection is here in its place. After resection of the joints there will be movements between the phalanges and metacarpi, and we are justified in making resection in yet existing or even beginning suppuration, to foster healing, etc. The small incision done early in the beginning of the panaritium, will save the surgeon from more mutilating cutting, and therefore the panaritium shows that minor but active surgery is great in its result.

